# How does artificial intelligence in radiology improve efficiency and health outcomes?

**DOI:** 10.1007/s00247-021-05114-8

**Published:** 2021-06-12

**Authors:** Kicky G. van Leeuwen, Maarten de Rooij, Steven Schalekamp, Bram van Ginneken, Matthieu J. C. M. Rutten

**Affiliations:** 1 Department of Medical Imaging, Radboud University Medical Center, P.O. Box 9101, 6500 HB Nijmegen, The Netherlands; 2 Department of Radiology, Jeroen Bosch Hospital, ‘s-Hertogenbosch, The Netherlands

**Keywords:** Artificial intelligence, Pediatrics, Evidence-based practice, Impact analysis, Innovation, Radiology, Value-based health care

## Abstract

Since the introduction of artificial intelligence (AI) in radiology, the promise has been that it will improve health care and reduce costs. Has AI been able to fulfill that promise? We describe six clinical objectives that can be supported by AI: a more efficient workflow, shortened reading time, a reduction of dose and contrast agents, earlier detection of disease, improved diagnostic accuracy and more personalized diagnostics. We provide examples of use cases including the available scientific evidence for its impact based on a hierarchical model of efficacy. We conclude that the market is still maturing and little is known about the contribution of AI to clinical practice. More real-world monitoring of AI in clinical practice is expected to aid in determining the value of AI and making informed decisions on development, procurement and reimbursement.

## Introduction

Artificial intelligence (AI) has the potential to change many aspects of health care. However, AI is a means, a tool, not the goal in itself. To create a positive impact on health care with this technology, the clinical goal should be clearly defined. The Da Vinci robot is a well-known example of innovative technology that became very popular very fast, but even today the cost-effectiveness and claim of improved patient outcomes are being debated [1, 2]. With health care expenses continuously rising through an increasingly older population and evolving technology, we should dare to be critical about what medical devices, including AI-based software, are actually improving health care or making it more efficient.

More than 150 AI products for radiology are on the market [3]. These products have been cleared by the Food and Drug Administration (FDA) or are European Conformity (CE) marked to allow clinical use in the United States and Europe, respectively. Although the supply is large, the scientific evidence on the validation and impact of these products remains limited [4, 5]. A study performed in 2020 showed that only 36 of 100 AI products analyzed had peer-reviewed evidence available on their efficacy [5].

The scientific evidence on the efficacy can be classified according to the hierarchical model developed by Fryback and Thornbury [6] back in 1991 to evaluate the contribution of diagnostic imaging to the patient management process. In a previous study this model was adapted (Table 1; [5, 6]) to be applicable to assess evidence on AI [5]. The lower levels describe the functioning and performance of the product (levels 1, 2). Evaluations regarding higher levels (levels 3–5) describe the impact on the diagnosis, therapy and outcome of the patient. Ultimately, level 6 evidence describes the impact of AI on a macro level, demonstrating the effects on costs and health.

**Table 1:** Hierarchical model of efficacy to assess the contribution of artificial intelligence (AI) software to the diagnostic imaging process, adapted from [5, 6]

Level	Explanation
Level 1t	Technical efficacy Study demonstrates the technical feasibility of the software
Level 1c	Potential clinical efficacy Study demonstrates the feasibility of the software to be clinically applied
Level 2	Diagnostic accuracy efficacy Study demonstrates the standalone performance of the software
Level 3	Diagnostic thinking efficacy Study demonstrates the added value to the diagnosis
Level 4	Therapeutic efficacy Study demonstrates the impact of the software on the patient management decisions
Level 5	Patient outcome efficacy Study demonstrates the impact of the software on patient outcomes
Level 6	Societal efficacy Study demonstrates the impact of the software on society by performing an economic analysis

Most studies demonstrated the accuracy of the algorithm (level 2), but (prospective) research showing the benefits in clinical practice (level 3 and up) was limited and covered only 18 of the 100 products evaluated [5]. Another recently published study systematically reviewed evidence on the economic impact of AI in health care (level 6) and found only six eligible articles [7], demonstrating the limited evidence of the impact of AI from a more global perspective.

**Fig. 1:**
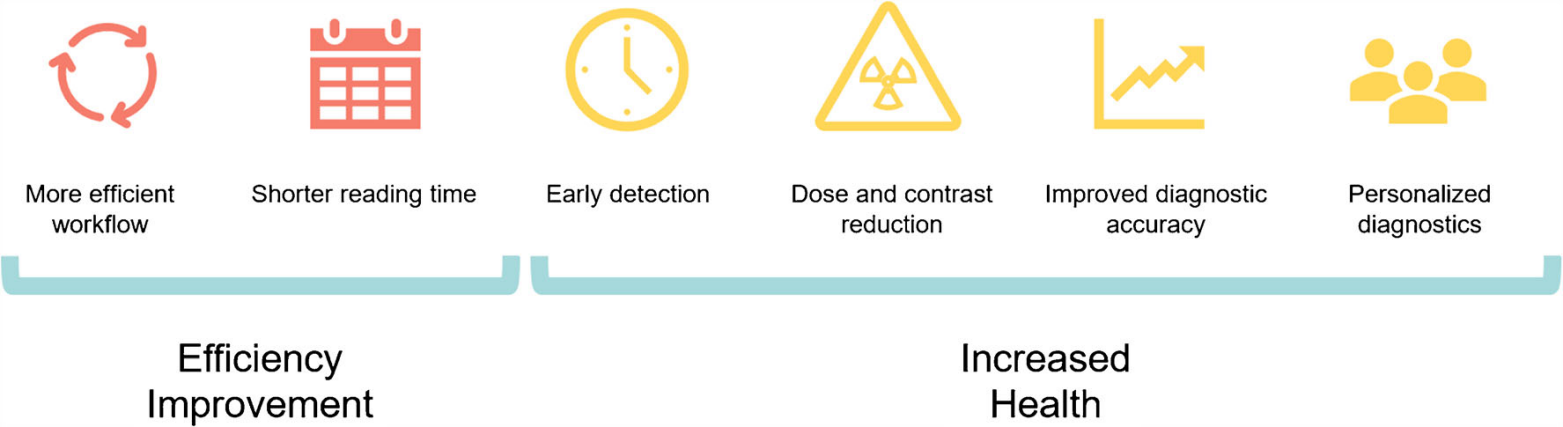
Six objectives that can be pursued with artificial intelligence in radiology to improve efficiency and health outcomes

Considering the framework of value-based health care and the corresponding value equation, value=outcome/cost, AI can create value when either reducing the costs or improving the health outcome [8]. We define these as the ultimate goals of AI in radiology, which can be supported by a variety of subgoals described as (1) making the workflow more efficient, (2) shortening the reading time, (3) reducing dose and contrast agents, (4) earlier detection of disease, (5) improved diagnostic accuracy and (6) more personalized diagnostics (Fig. 1).

In this paper, we expand upon the AI tasks and use cases supporting these six objectives, summarized in Table 2, and provide the evidence that demonstrates the (potential) impact of these solutions on health care.

**Table 2:** Overview of clinical objectives that can be reached with certain tasks performed by artificial intelligence (AI)-based software

Objectives →	Efficiency improvement		Increased health			
Tasks ↓	More efficient workflow	Shorter reading time	Early detection	Dose and contrast reduction	Improved diagnostic accuracy	Personalized diagnostics
Detection or diagnosis		Computer-aided detection; report generation	Incidental nodules; osteoporosis		Malignancies; abnormalities	Malignancy risk
Image enhancement	MR sequence synthesis	Bone suppression; vessel suppression		Low-dose CT; synthesized CT; pediatrics	Bone suppression; vessel suppression	
Image quality verification	Artifact detection; inadequate body position					
Image reconstruction				Low-dose CT; synthesized CT; pediatrics		
Quantitative analysis		Nodule size; brain volumetrics; bone age			Bone age; brain volumetrics; skeletal abnormalities	Breast density; brain volumetrics
Worklist prioritization			Critical findings; stroke; pneumothorax			
Worklist adaptation	Screening; normal filtering					
Scheduling	Appointments; no-shows; scanning protocols					

## Clinical objectives with artificial intelligence

### More efficient workflow

With ever increasing health care costs worldwide, effective use of the limited resources is an important endeavor. AI could contribute to this in clinical, but also non-clinical, ways. For example, even before a patient enters the radiology department, AI software might aid the scheduling of imaging appointments and predict no-shows for nudging or more efficient scheduling. Chong et al. [9] trained a model to predict which patients had the highest risk of missing their appointment. These patients received a phone call reminder, decreasing the no-show rate from 19.3% to 15.9% [9]. The impact of most of these solutions is not necessarily aimed at the detection or diagnosis of the patient; rather, these solutions address boundary conditions like patient management. The applications, therefore, involve lower risk and have fewer rules and regulations to comply to before they can be implemented in clinical practice. In spite of this, the availability and implementation of such software are limited, leaving room for growth of this industry.

The workflow might also be optimized by changing the diagnostic process with AI. A use case that is already widespread is AI software for tuberculosis detection on chest radiographs. AI-supported tuberculosis detection is especially useful in developing countries where staffing, expertise and financial resources are often limited. This can be used as an autonomous pre-screening tool to reduce the use of microbiological tests, which are more time-consuming and costly (levels 2, 3, 6) [10–13]. This is one of the first AI applications in radiology where the software functions autonomously and has taken over the task of the radiologist.

The potential of AI for workflow optimization is also being explored in use cases, but this has not been put into clinical practice. For mammography screening, for example, studies have been performed to simulate an alternative workflow in which an AI risk score determines the number of radiology reads (none, single or double), reducing the total amount of reading time (level 3) [14, 15]. For lung nodule detection on CT, it has been proposed to empower technicians with AI to leave only some of the workload to the radiologists (level 3) [16].

### Shorter reading time

Apart from increasing diagnostic accuracy and patient outcome, AI can contribute to increasing efficiency of the workforce. Between 2013 and 2018, CT and MR imaging exams increased by 54% and 48%, respectively, in the United Kingdom, while the radiology workforce grew only 19% [17]. Increasing numbers of diagnostic imaging examinations along with image technology improvements in the number of slices, reconstructions and sequences have resulted in many more images to review per patient. Decreasing the reading time of the exams could counteract this trend and potentially reduce the rate of burnout for pediatric radiologists as well [18].

Computer-aided detection (CAD) can decrease reading time by making the diagnostic process easier. Research has shown that reading time for normal cases decreased whereas the reading time for pathological cases slightly increased when using CAD tools [19]. Besides quality of the AI system, workflow integration is crucial for making this kind of software a success. Image enhancement could not only shorten image acquisition time but also ease detection, as shown by Martini et al. [20], who found that vessel suppression on CT thorax imaging resulted in a 21% reduction in reading time for the detection of pulmonary metastasis. Last, the automated quantification of nodules, brain volumes or other tissues, for example, might mitigate some of the tedious manual work that is part of a radiologist’s job, along with the large interrater variability inherent to these tasks [21, 22]. In pediatrics, the automated bone age prediction is a well adopted AI solution aiding the quantification and reading efficiency of hand radiographs [22, 23]. The device could be used autonomously, potentially reducing the reading time to zero, or as a concurrent read to speed quantification.

### Early detection

A timely diagnosis or intervention might be the objective to ultimately improve patient outcome. Especially in critical care situations, such as stroke diagnostics where the phrase “time is brain” is used, speed is important and AI has gained ground. AI software is used to analyze CTs and CT angiograms and notify radiologists, hub centers or even the intervention team directly when a large vessel occlusion or intracranial hemorrhage is present. Some preliminary prospective studies have shown the potential positive impact of AI in stroke care by reducing time between the CT angiography and the intervention from 281 min to 243 min, on average, and reducing length of stay (levels 4, 5) [24, 25].

An alternative method to reduce report turnaround times and promote early detection of critical findings includes worklist prioritization based on urgent findings detected by AI [26]. A study from a German university hospital simulated this concept on retrospective chest radiographs and found that turnaround times for reporting critical findings reduced from 80 min to 35–50 min [27]. In the United States, a commercial algorithm to prioritize intracranial hemorrhage resulted in reduced waiting time from 16 min to 12 min per positive case [28]. Apart from identifying urgent findings, incidental findings can also be detected early through AI notification. Examples are lung nodule detection on chest radiographs and automated detection of vertebral fractures to identify early signs of osteoporosis using AI applied to every chest or abdomen CT (level 2) [29].

### Dose and contrast reduction

A less renowned goal, but one that is very applicable for AI, is the reduction of radiation dose and intravenous contrast agents. This is even more relevant for pediatric patients because minimizing the use of radiation also minimizes the elevated risk of cancer in younger patients [30]. More frequently, deep learning is being used to advance and speed image reconstruction and post-processing [31, 32]. Such technology facilitates good image quality with a lower dose or even no dose, as shown by a study from Belgium in which commercial AI software was used to synthesize a CT from MR to assess lesions in the sacroiliac joints for the diagnosis of spondylarthritis, maintaining diagnostic accuracy and making CT potentially redundant (level 3) [33].

### Improved diagnostic accuracy

About half of the AI products for radiology on the market aim primarily at improving diagnostic accuracy [3] by increasing the sensitivity and/or specificity of the diagnostic test. These products are designed to decrease missed diagnoses or prevent unnecessary interventions or examinations, thereby improving health outcomes.

Computer-aided detection algorithms, which were around long before the rise of AI, serve this purpose the most. By reading the exam concurrently with the radiologist or as a second read, bounding boxes, markers and probability scores aid the radiologist in the diagnostic process. Many products are on the market and much research has been conducted to demonstrate the performance of these algorithms in comparison to radiologists or a ground truth (level 2) [13, 34–36]. However, most of these products cannot be used as a standalone medical device, and it is therefore the accuracy of the combination of the software and radiologist that matters (level 3). Considering bone age assessment, researchers demonstrated a substantial increase in diagnostic accuracy for two radiologists aided by AI software as opposed to using the Greulich-Pyle atlas only (level 3) [22].

Many CAD products are commercially available and have gained ground in clinical practice. These products focus on the detection of cancerous tissue such as breast lesions and lung nodules. However, computer-aided detection is not the only way to reach this objective. Image enhancement and quantitative analysis also support this goal. For example, it has been shown that bone or vessel suppression on thorax imaging can elevate the detection of lung nodules (level 3) [20, 37]. In the area of musculoskeletal radiology, we found that automated knee assessment might improve both the agreement between physicians and the accuracy of the osteoarthritis diagnosis (level 3) [38].

### Personalized diagnostics

Instead of acting based on knowledge and research of a population, AI algorithms can support precision medicine by predicting risks and outcomes based on individual characteristics. This can result in improved health outcomes and aid the allocation of resources, e.g., by providing treatment or additional testing to patients who are expected to benefit most. For example, in the field of neurology, AI-generated brain volume measurements were used to predict the need for further invasive testing to diagnose Alzheimer disease; by using this model, a high diagnostic accuracy could be achieved while only performing additional biomarker testing on 26% of the population (level 4) [39]. Similarly, for thyroid lesion assessment with US, researchers showed prospectively that biopsies could be avoided when the malignancy risk was assessed with the help of a commercially available AI tool (level 4) [40].

Within the context of breast cancer screening, the classification of breast density aims to personalize the screening process. It is known that women with dense breasts have a higher risk of cancer [41–43]. An automated method makes a stratified screening process feasible in which women with dense breasts receive screening more frequently or with other modalities such as MR imaging. A study from the Netherlands involving more than 40,000 women with extremely dense breast tissue according to commercially available AI software resulted in significantly fewer interval cancers in the group that received additional MR screening (level 5) [42].

The use of additional data such as clinical characteristics and genome information could enforce the ability to personalize predictions. However, this is not widespread within the commercially available AI-based radiology software.

## Discussion

**Fig. 2:**
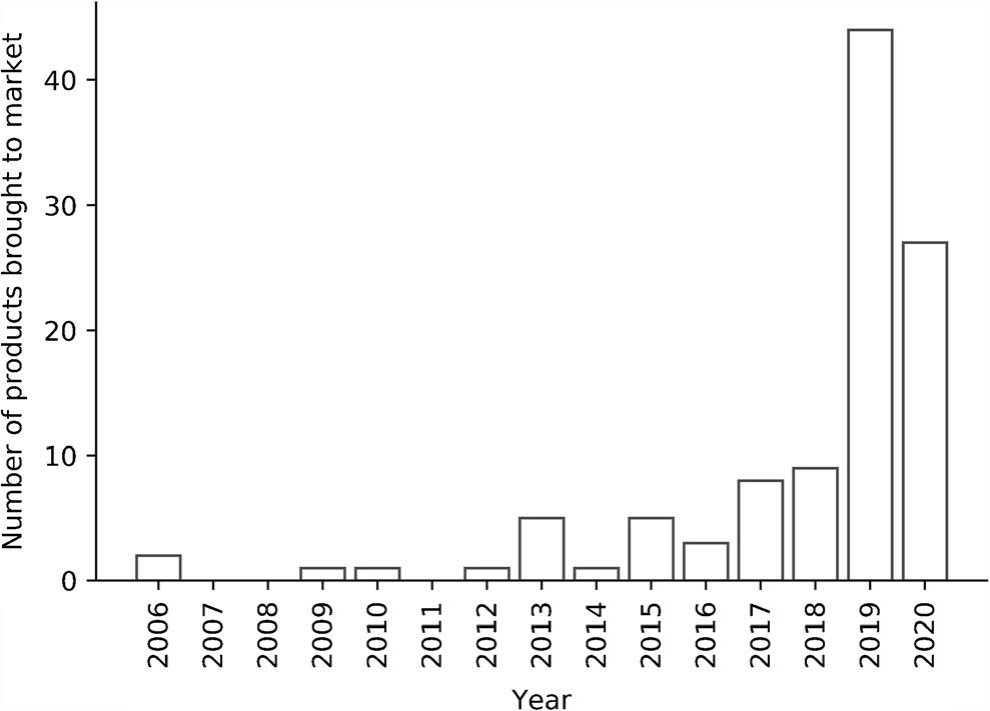
Number of artificial intelligence products in radiology brought to market based on data from [3]

Even though the potential of AI to create value to (pediatric) radiology and the patient management process is large, the impact has only been proved in a limited number of cases. Most evidence is based on simulations or retrospective studies. One of the reasons for the lack of evidence might be that the field is still maturing. Most products came to the market only in the last 2 years (Fig. 2) [3]. On average, it takes 17 years for health care innovations to become adopted in clinical practice [44]. Thus, one could argue that AI adoption in clinical practice is still in its infancy.

In the light of pediatric radiology, some applications such as bone age prediction have been widely adopted, but other use cases linger. Vendors tend to go for areas of application that are widely used, are relevant to a large population, and for which there is a large availability of training data (“low hanging fruit”). This causes significant overlap in the clinical use cases addressed by vendors, while many use cases remain unaddressed, including pediatric use cases.

Although FDA clearance and CE marking are present for many AI products, this does not guarantee the added clinical value. The notified bodies (Europe) and FDA (United States) assess the medical devices, including AI software, for risk of harm. Assessing the value and efficacy is up to the vendor and clients themselves, even more so considering that the efficacy is partly hospital-dependent because each hospital’s patient populations, imaging brands and workflows are different [45]. Therefore, it has been proposed by multiple parties, including the FDA, to change the way AI software is regulated from a standalone evaluation to a more systemic approach where the context of the clinical and human interaction is taken into account [45–47]. This would require more real-world monitoring contributing to the available evidence on the actual clinical impact of AI on health care.

Another challenge for creating clinical value with AI is the actual technical implementation and deployment of these algorithms. With many narrow task-oriented solutions on the market, the number of contracts and integrations can quickly proliferate and create a large overhead that does not contribute to optimizing efficiency. Further, the increase of information (biomarkers, quantifications, heatmaps, etc.) produced by the algorithms has to be presented in a way that does not decrease efficiency. Marketplaces and mediating platforms have emerged to take some of that burden away. These platforms are still in development and the added value both clinically and financially remains to be studied.

Considering the increased attention on value-based health care, determining the value of a new technology such as AI becomes more relevant for hospitals and insurance companies. With the abundance of AI tools, monitoring or even calculating the expected effects to make informed decisions is crucial. This might demonstrate the return of investment — quality or efficiency improvement — justifying the purchase. Monitoring efficacy and use might also minimize the presence of ghost software, or the situation in which software is being paid for but remains unused.

The question remains who is going to pay for the AI tools introduced. Insurance companies might have a role in reimbursing the costs of AI use. In 2002, approval for reimbursement was given in the United States for the use of CAD for mammography, which caused a swift increase in sales and use of the CAD systems [48]. However, this decision backfired as studies by Fenton et al. [49] in 2007 and by Lehman et al. [48] in 2015 showed that the use of these CAD systems in clinical practice did not improve detection rates. Now, the CAD for mammography can no longer be reimbursed separately, making it only profitable to use if the system really improves the efficiency or quality of the tumor detection.

In 2020, Medicare and Medicaid Services took another approach to support the financing and adoption of AI tools in acute stroke using the New Technology Add-on Payment program [50]. The AI solutions aid in the detection of large-vessel occlusions in acute stroke and alert the stroke team to decrease the time to treatment. Preliminary studies have shown that patients might experience less disability and are thus potentially in less need of extra care later in life [24]. These potential benefits mostly lie in the long term, although the costs for the software are made in the short term by, for example, the radiology department. Insurance coverage could be a game changer in AI adoption but requires profound evidence of the clinical impact of the AI tool and use case. Time will tell whether this new approval has (re-)opened the gates for other clinical use cases in- and outside the United States.

## Conclusion

Even though the potential of AI software to impact radiology is large, little is known about how it is changing the quality, efficiency and costs of health care. History has shown that real-world validation of these innovations is essential to making informed decisions on further development, procurement, implementation and reimbursement.

Closely evaluating and monitoring the experiences and impact of the AI products in clinical practice should provide insights in its contribution to the initial health care improvement goals. Only then can we prove whether AI is contributing to improved health care with respect to both costs and health outcomes.
